# Predictors of in-hospital mortality after mitral valve surgery for post-myocardial infarction papillary muscle rupture

**DOI:** 10.1186/s13019-014-0171-z

**Published:** 2014-10-18

**Authors:** Wobbe Bouma, Inez J Wijdh-den Hamer, Bart M Koene, Michiel Kuijpers, Ehsan Natour, Michiel E Erasmus, Iwan C C van der Horst, Joseph H Gorman III, Robert C Gorman, Massimo A Mariani

**Affiliations:** Department of Cardiothoracic Surgery, University of Groningen, University Medical Center Groningen, Groningen, The Netherlands; Department of Critical Care, University of Groningen, University Medical Center Groningen, Groningen, The Netherlands; Gorman Cardiovascular Research Group, University of Pennsylvania, Hospital of the University of Pennsylvania, Philadelphia, PA USA

**Keywords:** Myocardial infarction, Papillary muscle (rupture), Mitral regurgitation, Mitral valve repair, Mitral valve replacement, Outcome

## Abstract

**Background:**

Papillary muscle rupture (PMR) is a rare, but often life-threatening mechanical complication of myocardial infarction (MI). Immediate surgical intervention is considered the optimal and most rational treatment for acute PMR, but carries high risks. At this point it is not entirely clear which patients are at highest risk. In this study we sought to determine in-hospital mortality and its predictors for patients who underwent mitral valve surgery for post-MI PMR.

**Methods:**

Between January 1990 and December 2012, 48 consecutive patients (mean age 64.9 ± 10.8 years) underwent mitral valve repair (n = 10) or replacement (n = 38) for post-MI PMR. Clinical data, echocardiographic data, catheterization data, and surgical reports were reviewed. Univariate and multivariate logistic regression analyses were performed to identify predictors of in-hospital mortality.

**Results:**

Intraoperative mortality was 4.2% and in-hospital mortality was 25.0%. Univariate and multivariate logistic regression analyses revealed the logistic EuroSCORE and EuroSCORE II as independent predictors of in-hospital mortality. Receiver operating characteristics curves showed an optimal cutoff value of 40% for the logistic EuroSCORE (area under the curve 0.85, 95% CI 0.71-1.00, *P* < 0.001) and of 25% for the EuroSCORE II (area under the curve 0.83, 95% CI 0.68-0.99, *P* = 0.001). After removal of the EuroSCOREs from the model, complete PMR and intraoperative intra-aortic balloon pump (IABP) requirement were independent predictors of in-hospital mortality.

**Conclusions:**

The logistic EuroSCORE (optimal cutoff ≥40%), EuroSCORE II (optimal cutoff ≥25%), complete PMR, and intraoperative IABP requirement are strong independent predictors of in-hospital mortality in patients undergoing mitral valve surgery for post-MI PMR. These predictors may aid in surgical decision making and they may help improve the quality of informed consent.

**Electronic supplementary material:**

The online version of this article (doi:10.1186/s13019-014-0171-z) contains supplementary material, which is available to authorized users.

## Background

Papillary muscle rupture (PMR) is a rare, but often life-threatening mechanical complication of myocardial infarction (MI) [1]. It occurs in 1% to 5% of patients with acute MI and accounts for 5% of infarct-related deaths [1],[2]. Approximately 80% of ruptures occur within 7 days after MI, but a delayed rupture several weeks or months after MI is also possible [1]-[3]. When treated only medically, mortality may be as high as 50% in the first 24 hours (especially when PMR is complete), and as high as 80% in the first week [3],[4]. Since the first mitral valve replacement (MVR) for post-MI PMR in 1965 [5], several reports have emphasized that immediate surgical intervention is the optimal and most rational treatment for acute PMR, despite high risks [2],[3],[6],[7]. Although mitral valve repair may lead to a better outcome due to a better preservation of postoperative left ventricular function [8]-[13], MVR is generally preferred in these unstable, high-risk patients [7],[14]-[17]. Because of the high risk some surgeons may be reluctant to operate these patients, while others are willing to accept the high risk. At this point it is not entirely clear which patients are at highest risk.

We present one of the largest series to date of patients who underwent mitral valve surgery for post-MI PMR. In this study we sought to determine in-hospital mortality and its predictors. Identifying these predictors may aid the surgical decision making process and it may help improve the quality of informed consent.

## Methods

This study was conducted in accordance with the guidelines of the University Medical Center Groningen Institutional Review Board.

### Patients

Between January 1990 and December 2012, 48 consecutive patients underwent mitral valve surgery for moderate (grade 3+) or severe (grade 4+) mitral regurgitation (MR) caused by post-MI PMR. Baseline patient characteristics are summarized in Table 1. All patients had a documented MI before PMR. Infarct location was determined electrocardiographically and echocardiographically (by the detection of wall motion abnormalities).

**Table 1:** **Preoperative patient data (n = 48)**

Variable^a^		Value
Age, years		64.9 ± 10.8
Gender		
Male		34 (71)
Female		14 (29)
NYHA functional class		
Class III		7 (15)
Class IV		41 (85)
EuroSCORE I (logistic),%		29.4 ± 22.7
EuroSCORE II,%		19.4 ± 14.6
Previous myocardial infarction		48 (100)
Inferior and/or posterior		32 (67)
Inferoposterolateral		13 (27)
Anterolateral		9 (19)
Coronary artery disease		48 (100)
Left main stenosis		3 (6)
One-vessel disease		23 (48)
Two-vessel disease		17 (35)
Three-vessel disease		8 (17)
Infarct related artery		
Left anterior descending coronary artery		1 (2)
Left circumflex coronary artery		28 (58)
Right coronary artery		19 (40)
Previous percutaneous coronary intervention		12 (25)
Previous cardiac surgery		0 (0)
Preoperative grade of mitral regurgitation		
3+ (moderate)		1 (2)
4+ (severe)		47 (98)
Preoperative LV function		
Normal	(EF >50%)	34 (71)
Moderately impaired	(EF 30-50%)	10 (21)
Severely impaired	(EF <30%)	4 (8)
Heart rhythm		
Sinus rhythm		43 (90)
Atrial fibrillation		5 (10)
Pacemaker		0 (0)
Pulmonary artery pressure		
Systolic/diastolic, mmHg		46 ± 13/25 ± 10
Mean, mmHg		32 ± 10
Pulmonary capillary wedge pressure, mmHg		24 ± 14
Mechanical ventilation		23 (48)
Inotropic drug support		26 (54)
Intra-aortic balloon pump		21 (44)
Serum creatinine, μmol/L		162 ± 100
Acute renal failure		10 (21)
Cardiogenic shock		31 (65)
History of congestive heart failure		4 (8)
Hypertension		11 (23)
Diabetes mellitus		9 (19)
Smoking		16 (33)
Hypercholesterolemia		5 (10)
Obesity (body mass index >30 kg/m^2^)		7 (15)
Peripheral vascular disease		2 (4)
Family history of coronary artery disease		6 (13)
Chronic renal disease		1 (2)
Chronic obstructive pulmonary disease		2 (4)
Cerebrovascular disease		5 (10)

^a^Data are presented as mean ± standard deviation or number (%).

EF: ejection fraction; LV: left ventricle; NYHA: New York Heart Association.

Clinical data, echocardiographic data, catheterization data, and surgical reports were reviewed. Intraoperative mortality was defined as death during surgery. In-hospital mortality was defined as death during surgery, within 30 days after surgery or during the same hospital stay. Follow-up was complete.

### Echocardiography and coronary angiography

All patients underwent preoperative echocardiography (transthoracic (TTE) and/or transesophageal (TEE)) and coronary angiography. TTE accurately revealed the diagnosis of PMR in 18 patients. PMR was suspected in the remaining 30 patients and confirmed with TEE in 24 patients. In 6 patients the exact mechanism of MR remained inconclusive. Left ventricular function was assessed by echocardiography. In addition, wall motion abnormalities were documented for infarct localization.

### Surgical technique

Surgical data is summarized in Table 2.

**Table 2:** **Surgical data (n = 48)**

Variable^a^	Value
Mitral valve surgery	
Salvage	2 (4)
Emergent	29 (60)
Urgent	11 (23)
Elective	6 (13)
Timing of mitral valve surgery	
Surgery ≤7 days after MI	27 (56)
Surgery >7 days and ≤30 days after MI	9 (19)
Surgery >30 days after MI	12 (25)
Posteromedian papillary muscle rupture	42 (88)
Complete	15 (36)
Incomplete	2 (5)
Partial	25 (59)
Anterolateral papillary muscle rupture	5 (10)
Complete	4 (80)
Incomplete	0 (0)
Partial	1 (20)
PMPM and ALPM rupture (both complete)	1 (2)
Leaflet prolapse	
AMVL prolapse	12 (25)
PMVL prolapse	15 (31)
AMVL and PMVL prolapse	21 (44)
Surgical approach	
Left atriotomy	34 (71)
Transseptal	13 (27)
Left ventriculotomy	1 (2)
Mitral valve replacement	38 (79)
Mmechanical prosthesis	35 (92)
Bioprosthesis	3 (8)
(partial) preservation of the subvalvular apparatus	24 (63)
Mitral valve repair	10 (21)
Reimplantation of the PM in the LV wall and annuloplasty ring (Carpentier-Edwards Classic)	1 (10)
Reimplantation of the PM in the corresponding PM with a sandwiched pledget-reinforced PTFE suture and annuloplasty ring (Carpentier-edwards Classic or Physio II	2 (20)
Quadrangular resection of P2 and annuloplasty ring (Carpentier-Edwards Classic or Carbomedics)^b^	6 (60)
Commissuroplasty and annuloplasty ring (Carpentier- Edwards Physio II)	1 (10)
Intraoperative mitral valve repair failure	1 (9)
Concomitant surgery	28 (58)
Coronary artery bypass grafting	24 (50)
Septal rupture closure	2 (4)
Aortic valve replacement	1 (2)
Tricuspid valve plasty	2 (4)
Duration of surgery, min	278 ± 88
Cardiopulmonary bypass time, min	178 ± 68
Aortic cross-clamp time, min	98 ± 36
Intraoperative IABP requirement	24 (50)

^a^Data are presented as mean ± standard deviation or number (%).

^b^Failed in 1 patient intraoperatively and resulted in mitral valve replacement (not counted as mitral valve repair).

ALPM: anterolateral papillary muscle; AMVL: anterior mitral valve leaflet; IABP: intra-aortic balloon pump; LA: left atrium; LV: left ventricle; MI: myocardial infarction; PMPM: posteromedian papillary muscle; PM(R): papillary muscle (rupture); PMVL: posterior mitral valve leaflet; PTFE: polytetrafluorethylene.

Patients were considered to undergo a salvage operation when brought to the operating room under cardiopulmonary resuscitation, an emergency operation when brought to the operating room directly from the catheterization lab or intensive care unit because of haemodynamic instability, and an urgent operation when operated on during the same hospitalization as for angiography because their discharge was deemed medically unreasonable [18]. Otherwise the operation was considered elective.

PMR was confirmed during surgery in all patients. When a papillary muscle (PM) was divided into several heads, rupture of a single head was defined as “partial” [12],[13]. In case of detachment of the main insertion of a head which still remained fixed to the remnant PM via muscular bridges, rupture was defined as “incomplete” [12],[13]. Rupture of the whole PM was defined as “total and complete” [12],[13].

Myocardial protection was carried out using moderate systemic hypothermia and antegrade or combined antegrade and retrograde cardioplegia. The mitral valve was exposed with a left atriotomy or a transseptal approach (or with a left ventriculotomy in one patient with a ventricular septal rupture). Surgeon’s choice dictated treatment strategy. Repair techniques are shown in Table 2. Concomitant procedures were performed in 28 patients and concomitant coronary artery bypass grafting (CABG) was performed in 24 patients (Table 2). After weaning from cardiopulmonary bypass mitral valve competence was confirmed with TEE.

Patients who underwent mitral valve repair or MVR with a bioprosthesis received acenocoumarol treatment for 3 months and patients who underwent MVR with a mechanical prosthesis were put on lifelong acenocoumarol treatment. In addition, patients who underwent concomitant CABG also received lifelong acetylsalicylic acid treatment.

### Statistics

Continuous variables were expressed as mean ± standard deviation. Categorical variables were expressed as percentages. Comparisons between groups for univariate analysis of in-hospital mortality were performed using Pearson`s χ^2^ test or Fisher’s exact test (two-sided) as appropriate for categorical variables and the independent samples *t*-test or Mann–Whitney U test (two-sided) as appropriate for continuous variables. Univariate variables with *P* < 0.10 were included in the multivariate analysis. Age and gender were included in all multivariate models, irrespective of the results of univariate analysis. Multivariate analyses were performed with the logistic EuroSCORE (model 1), with the EuroSCORE II (model 2), and without the EuroSCOREs (model 3). Multivariate logistic regression analyses by means of a forward stepwise algorithm (cutoff for entry and removal set at a significance level of 0.05) were performed to identify independent predictors of in-hospital mortality. Odds ratios were reported with 95% confidence intervals (CI). Goodness-of-fit of the final logistic regression models was assessed with the Hosmer-Lemeshow statistic.

Receiver operating characteristic (ROC) curves were calculated for continuous independent predictors to single out the optimal cutoff value of predicting in-hospital mortality. The point with the largest sum of sensitivity and specificity was chosen as a threshold. The area under the curve (AUC) was estimated by the non-parametric Wilcoxon-Mann–Whitney U statistic and standard error (SE) was calculated with the method of DeLong and Clarke-Pearson [19]. The statistical significance of difference of AUC from the “no discrimination line” was evaluated by the Mann–Whitney U statistic.

All calculations were performed using commercially available statistical packages (IBM SPSS Statistics 21.0; IBM Corporation, Chicago, IL, USA and Stats Direct 2.8.0; StatsDirect Ltd, Cheshire, UK). Statistically significant differences were established at *P* < 0.05.

## Results

### In-hospital mortality

Two patients died during surgery (intraoperative mortality rate of 4.2%). One patient could not be weaned from cardiopulmonary bypass and the other patient died due to heart failure directly after weaning from cardiopulmonary bypass. In addition to two intra-operative deaths, there were another 10 postoperative deaths. In-hospital mortality was 25.0%. Causes of in-hospital death are shown in Table 3.

**Table 3:** **Postoperative patient data (n = 48)**

Variable/Condition^a^		Value
Intraoperative mortality		2 (4)
Immediate postoperative grade of MR (TEE) (n = 46^b^)		
0 (no or trace)		44 (96)
1+ (trivial)		2 (4)
Postoperative morbidity (n = 46^b^)		
Re-exploration for bleeding		5 (11)
Re-exploration for cardiac tamponade		3 (7)
Prolonged inotropic support (>24 hours)		22 (48)
Prolonged respiratory support (>24 hours)		19 (41)
Post-operative hemodialysis		7 (15)
In-hospital mortality^c^		12 (25)
Causes of in-hospital death (n = 12)^c^		
Heart failure (unable to wean from CPB)		1 (8)
Heart failure		7 (58)
Septal rupture		1 (8)
Left ventricular rupture		1 (8)
Haemorrhagic shock (massive bleeding)		2 (17)
Total hospital stay, days		18.7 ± 15.5
Intensive care unit stay, days		9.7 ± 11.0

^a^Data are presented as mean ± standard deviation or number (%).

^b^Number of patients at risk left.

^c^Includes intraoperative deaths.

CPB: cardiopulmonary bypass; MR: mitral regurgitation; TEE: trans-esophageal echocardiography.

### Predictors of in-hospital mortality

Univariate and multivariate logistic regression analyses of in-hospital mortality are shown in Table 4. Multivariate analyses were performed with the logistic EuroSCORE (model 1), with the EuroSCORE II (model 2), and without the EuroSCOREs (model 3).

**Table 4:** **Predictors of in-hospital mortality by univariate analysis and multivariate logistic regression analysis**

	Univariate analysis			Multivariate analysis		
Variable	OR	95% CI	***P***value	OR	95% CI	***P***value
Logistic EuroSCORE, %	1.08	(1.03-1.12)	<0.001	1.07	(1.03-1.12)	0.002^a^
EuroSCORE II, %	1.12	(1.04-1.21)	0.001	1.12	(1.04-1.21)	0.003^b^
Preoperative LVEF <30%	11.67	(1.08-125.90)	0.043	−	−	−
Mechanical ventilation	4.71	(1.09-20.47)	0.030	−	−	−
Preoperative inotropic drug support	7.00	(1.34-36.69)	0.012	−	−	−
Acute renal failure	4.43	(1.00-19.58)	0.094	−	−	−
Cardiogenic shock	8.80	(1.03-75.55)	0.035	−	−	−
Salvage or emergent mitral valve surgery	8.80	(1.03-75.55)	0.035	−	−	−
Complete AL or PM PMR	4.55	(1.13-18.32)	0.041	6.51	(1.18-35.78)	0.031^c^
Mitral valve replacement	9.91	(0.54-182.88)	0.048	−	−	−
MVR without preservation of thesubvalvular apparatus	5.80	(1.41-23.84)	0.024	−	−	−
Cardiopulmonary bypass time, min	1.01	(1.00-1.02)	0.036	−	−	−
Intraoperative IABP requirement	19.46	(2.25-168.27)	0.001	18.70	(1.96-178.79)	0.011^c^

^a^Model 1; ^b^Model 2; ^c^Model 3.

AL: anterolateral; CI: confidence interval; IABP: intra-aortic balloon pump; LVEF: left ventricular ejection fraction; MVR: mitral valve replacement; OR: odds ratio; PM: posteromedian; PMR: papillary muscle rupture.

#### Model 1

**Figure 1:**
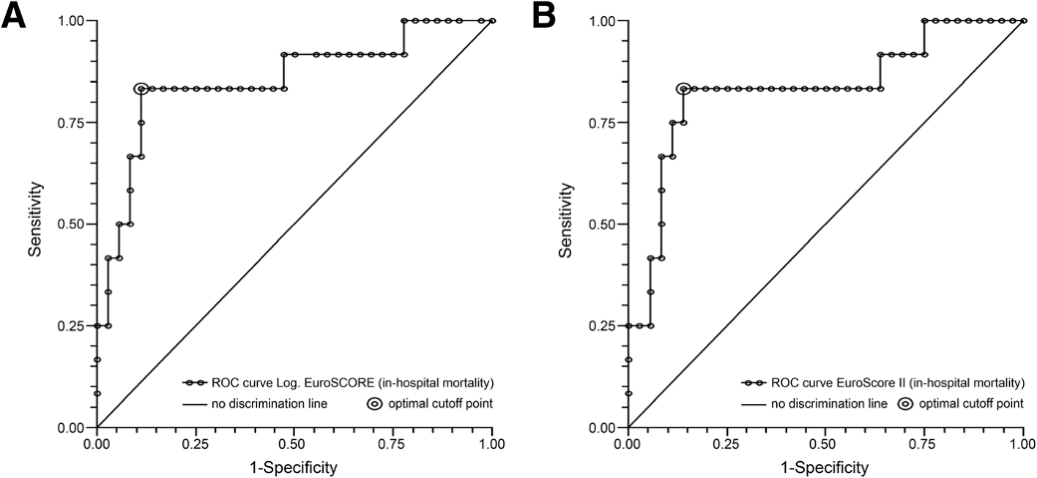
**Receiver operating characteristic (ROC) curves. A**: ROC curve for the logistic EuroSCORE as a predictor of in-hospital mortality (optimal cutoff value 40%). **B**: ROC curve for the EuroSCORE II as a predictor of in-hospital mortality (optimal cutoff value 25%).

Multivariate analysis with the logistic EuroSCORE revealed the logistic EuroSCORE as an independent predictor of in-hospital mortality (odds ratio 1.07 (95% CI 1.03-1.12), Wald χ^2^ 9.34, *P* = 0.002). The Hosmer-Lemeshow goodness-of-fit test was non-significant, indicating that this multivariate model is a good fit (χ^2^ = 6.72, df = 7, *P* = 0.459). A receiver operating characteristic (ROC) curve was calculated for the logistic EuroSCORE to single out the optimal cutoff value of predicting in-hospital mortality (Figure 1A). The optimal cutoff value was 40% with an area under the curve (AUC) of 0.85 (95% CI 0.71-1.00, *P* < 0.001), a sensitivity of 83.3% and a specificity of 88.9%.

#### Model 2

Multivariate analysis with the EuroSCORE II revealed the EuroSCORE II as an independent predictor of in-hospital mortality (odds ratio 1.12 (95% CI 1.04-1.21), Wald χ^2^ 8.82, *P* = 0.003). The Hosmer-Lemeshow goodness-of-fit test was non-significant, indicating that this multivariate model is a good fit (χ^2^ = 9.42, df = 7, *P* = 0.224). A ROC curve was calculated for the EuroSCORE II to single out the optimal cutoff value of predicting in-hospital mortality (Figure 1B). The optimal cutoff value was 25% with an AUC of 0.83 (95% CI 0.68-0.99, *P* = 0.001), a sensitivity of 83.3% and a specificity of 86.1%.

#### Model 3

After removal of the EuroSCOREs from the model, complete papillary muscle rupture (odds ratio 6.51 (95% CI 1.18-35.78), Wald χ^2^ 4.64, *P* = 0.031) and intra-operative IABP requirement (odds ratio 18.70 (95% CI 1.96-178.79), Wald χ^2^ 6.46, *P* = 0.011) were independent predictors of in-hospital mortality. The Hosmer-Lemeshow goodness-of-fit test was non-significant, indicating that this multivariate model is a good fit (χ^2^ = 0.40, df = 2, *P* = 0.818).

## Discussion

PMR is a rare, but serious mechanical complication of MI, which can lead to rapid clinical deterioration and death [1]-[4],[6],[7]. Immediate surgical intervention is considered the optimal and most rational treatment for acute PMR, but it still carries high risks [2],[3],[6],[7]. In this study intra-operative mortality was 4.2% and in-hospital mortality was 25.0%. Other studies have shown similar short-term mortality rates with intra-operative mortality ranging between 0 and 6% and in-hospital mortality ranging between 19 and 39% [7],[14]-[17],[20].

The logistic EuroSCORE and EuroSCORE II were important independent predictors of in-hospital mortality in this study. The logistic EuroSCORE is an important risk-stratification model in cardiac surgery which was introduced in 1999 [21]. The model was mainly designed for predicting in-hospital mortality in CABG patients. In the past decade the predictive power of the logistic EuroSCORE has proven its value in research, clinical practice and quality monitoring. In recent years, however, the predictive power of the logistic EuroSCORE has declined (probably due to changes in populations and improvement in surgical techniques and perioperative care) with a relative overestimation of in-hospital mortality in low-risk patients and a relative underestimation in high-risk patients [22]. To more accurately predict in-hospital mortality for patients undergoing a wider range of contemporary cardiac surgical procedures the EuroSCORE II was introduced in 2012 [23]. In this study the mean logistic EuroSCORE was 29.4% and the mean EuroSCORE II was 19.4%, which means that the logistic EuroSCORE overestimated and the EuroSCORE II underestimated the actual in-hospital mortality rate in this cohort (25.0%). Although both EuroSCOREs were not specifically designed for patients undergoing mitral valve surgery for post-MI PMR, these models can be used to predict in-hospital mortality in this setting. ROC curve analysis showed that the optimal cutoff value for reliably predicting in-hospital mortality was 40% for the logistic EuroSCORE and 25% for EuroSCORE II.

The posteromedian papillary muscle (PMPM) ruptures 3–12 times more frequently than the anterolateral papillary muscle (ALPM) after MI [2],[3],[6],[7]. The ALPM is less vulnerable to rupture due to its dual blood supply from the left anterior descending coronary artery and circumflex coronary artery [24]. The PMPM is more prone to ischemia and rupture due to its dependence on single blood supply from the posterior descending coronary artery (which is either derived from the circumflex or from the right coronary artery) [24]. In this study PMPM rupture occurred in 42 patients (88%) and ALPM rupture occurred in 5 patients (10%), which supports these previous findings. Double PMR occurred in 1 patient (2%). This study shows that ALPM rupture is usually complete (80% of cases). PMPM rupture is usually partial (59% of cases), due to the fact that the PMPM is frequently subdivided into several heads [12],[13]. Partial PMR can lead to varying degrees of mitral regurgitation, but complete PMR causes prolapse of both the anterior and posterior leaflet and severe mitral regurgitation. Complete PMR usually results in a more critical preoperative state with imminent haemodynamic instability and cardiogenic shock. In-hospital mortality was 42.1% for patients with complete PMR and 13.8% for patients with partial or incomplete PMR. Complete PMR was an independent predictor of in-hospital mortality in this study.

Preoperative haemodynamic instability and cardiogenic shock or difficulty to wean from CPB may warrant the use of an intra-aortic balloon pump (IABP) to offload the left ventricle by reducing afterload and improving coronary perfusion [25]. Preoperative stabilization should not lead to a false sense of security and delay of surgery, because the subsequent course can be characterized by sudden and unpredictable deterioration and progression to death [2],[3],[6],[7]. An IABP was required in 21 patients (44%) preoperatively. The IABP could be removed in 3 patients at the end of the procedure. Another 6 patients required an IABP intraoperatively. Therefore, intraoperative IABP requirement was 50%. Similar to the findings reported in several other studies [14],[17], preoperative IABP requirement was not a predictor of in-hospital mortality in this study. Intraoperative IABP requirement, however, was a strong independent predictor of in-hospital mortality; with an in-hospital mortality of 45.8% for patients who required an IABP intraoperatively versus an in-hospital mortality of 4.0% for patients who did not require an IABP intraoperatively, *P* = 0.001. This finding highlights the negative impact of immediate postoperative haemodynamic instability on in-hospital mortality.

Although PMR can lead to rapid haemodynamic instability, left ventricular ejection fraction (LVEF) appears to be relatively preserved in patients requiring mitral valve surgery for post-MI PMR [7],[14]. The data from this study supports that finding, since 71% of patients had a normal LVEF (>50%), 21% had a moderately impaired LVEF (30-50%), and only 8% had a severely impaired LVEF (<30%). LVEF did not predict in-hospital mortality in our study and several other studies [14],[17]. A relatively preserved LVEF may result in greater shearing forces on the ischemic PM, which may render it more prone to rupture [3]. Preservation of LVEF may be related to limited infarct size [7] and limited extent of coronary artery disease (CAD) in this population [7],[14]. Different studies have shown a high incidence of one-vessel CAD in patients requiring mitral valve surgery for post-MI PMR, ranging from 23% to 44% [7],[14]. This study supports these findings with an incidence of one-vessel CAD of 48%.

The additional benefit of concomitant CABG in the setting of mitral valve surgery for post-MI PMR remains unclear. Two studies from the Mayo Clinic have shown that concomitant CABG can improve immediate and long-term survival [7],[16]. Similar to the findings reported in several other studies [15],[17], concomitant CABG was not a predictor of in-hospital mortality in this study. In-hospital mortality was 20.8% in the concomitant CABG group and 29.2% in the no concomitant CABG group, *P* = 0.505. We also determined if revascularization (with a preoperative percutaneous coronary intervention (PCI) and/or with concomitant CABG) predicted in-hospital mortality in patients undergoing mitral valve surgery for post-MI PMR. 12 patients underwent a preoperative PCI (balloon angioplasty with or without stenting); 24 patients underwent concomitant CABG (4 of these patients had also undergone a preoperative PCI). In-hospital mortality was 21.9% in the revascularization group and 31.3% in the no revascularization group, *P* = 0.500. Revascularization did not predict in-hospital mortality. At this point, there are no randomized studies to determine the importance of concomitant CABG in this setting. Concomitant CABG may improve postoperative LV function and survival, but these potential benefits have to be weighed against the consequences of prolonging the duration of cardiopulmonary bypass (CPB). CPB time was a predictor of in-hospital mortality (but not an independent predictor) and mean CPB time was significantly longer in patients who underwent concomitant CABG (207 minutes) versus no concomitant CABG (149 minutes), *P* = 0.002. A hybrid approach with mitral valve surgery followed by PCI, if required, or preoperative PCI of the infarct-related artery (culprit lesion) followed by mitral valve surgery might be useful alternative strategies. Especially because the percentage of patients with single vessel CAD is high in this population; 48% in our study and 23-44% in other studies [7],[14]. Randomized studies would have to identify whether such hybrid approaches are superior to concomitant CABG in the setting of post-MI PMR.

When post-MI PMR is complete, repair is often not possible or advisable because of friable infarcted tissue [7],[12],[15],[16]. Mitral regurgitation secondary to partial or incomplete PMR with limited adjacent tissue damage is often amenable to a reliable repair, provided established repair techniques are used and adjacent tissue is not friable [7],[8],[10]-[13],[16],[26]. All 10 repair patients in this study experienced partial or incomplete PMR. (Ischemic) PMR is one of the rare conditions in which several Carpentier functional types of regurgitation (type I, annular dilatation, type II leaflet prolapse, and type IIIb restricted leaflet motion) can more or less coexist [26], which is important to realize during repair. In-hospital mortality was 0.0% in the repair group versus 31.6% in the replacement group, *P* = 0.048. MVR was not an independent predictor of in-hospital mortality. MVR without preservation of the subvalvular apparatus (i.e. with disruption of valvular-ventricular or papillary muscle-annular continuity) was associated with a higher in-hospital mortality (50.0% for MVR without preservation of the subvalvular apparatus versus 20.8% for MVR with preservation of the subvalvular apparatus, *P* = 0.081; or versus 14.7% for the combined group with a preserved subvalvular apparatus (repair or MVR with preservation of the subvalvular apparatus), *P* = 0.024), but it was not an independent predictor of in-hospital mortality. In general it has been shown that MVR with preservation of the subvalvular apparatus maintains postoperative LV contractile function and improves outcome [9]. As shown in this study, preservation of the subvalvular apparatus in MVR for post-MI PMR also seems to have a beneficial influence on in-hospital mortality.

Limitations of this study include the retrospective design, the long time frame, and the relatively small number of patients. However, compared to the previously published literature on this subject this is one of the largest published cohorts that underwent mitral valve surgery for post-MI PMR. Future (multicenter) investigations should include larger (preferably randomized) cohorts to more accurately identify independent predictors of short- and long-term outcome and to determine outcome benefits of mitral valve repair versus replacement for post-MI PMR.

## Conclusions

Our findings indicate that the logistic EuroSCORE (optimal cutoff ≥40%), EuroSCORE II (optimal cutoff ≥25%), complete PMR, and intraoperative IABP requirement are strong independent predictors of in-hospital mortality in patients undergoing mitral valve surgery for post-MI PMR. These predictors may aid in surgical decision making and they may help improve the quality of informed consent.

## Authors’ original submitted files for images

Below are the links to the authors’ original submitted files for images. Authors’ original file for figure 1

## Abbreviations

ALPM: Anterolateral papillary muscle
AMVL: Anterior mitral valve leaflet
AUC: Area under the curve
CABG: Coronary artery bypass grafting
CAD: Coronary artery disease
CI: Confidence interval
CPB: Cardiopulmonary bypass
IABP: Intra-aortic balloon pump
LA: Left atrium
LV(EF): Left ventricular (ejection fraction)
MI: Myocardial infarction
MR: Mitral regurgitation
MVR: Mitral valve replacement
NYHA: New-York Heart Association
OR: Odds ratio
PCI: Percutaneous coronary intervention
PMPM: Posteromedian papillary muscle
PM(R): Papillary muscle (rupture)
PMVL: Posterior mitral valve leaflet
PTFE: Polytetrafluorethylene
ROC: Receiver operating characteristic
SE: Standard error
TEE: Transesophageal echocardiography
TTE: Transthoracic echocardiography
