# Supplementary figures and images for: Predictors of in-hospital mortality after mitral valve surgery for post-myocardial infarction papillary muscle rupture
Source: J Cardiothorac Surg. 2014 Oct 18;9:171. doi: 10.1186/s13019-014-0171-z (PMC4201923; doi:10.1186/s13019-014-0171-z)

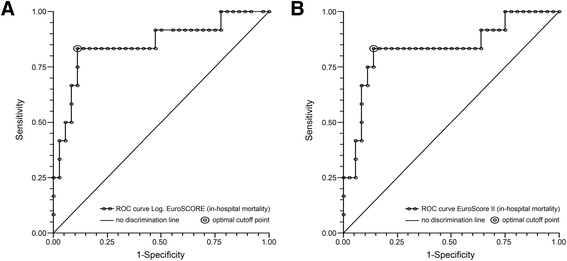

Supplement: Supplementary file 1 — Authors’ original file for figure 1 [file 13019_2014_171_MOESM1_ESM.gif]
